# Fabrication and characterization of Ni-decorated h-BN powders with ChCl-EG ionic liquid as addition by electroless deposition

**DOI:** 10.1098/rsos.180146

**Published:** 2018-05-02

**Authors:** Qionglian Yang, Juanjian Ru, Peng Song, Mingyu Hu, Jing Feng

**Affiliations:** 1 Faculty of Materials Science and Engineering, Kunming University of Science and Technology, Kunming 650093, People's Republic of China; 2 Faculty of Metallurgical and Energy Engineering, Kunming University of Science and Technology, Kunming 650093, People's Republic of China

**Keywords:** ceramics, electroless deposition, h-BN powders, ionic liquid, nickel plating times, deposition mechanism

## Abstract

Ni-decorated h-BN powders are fabricated with ChCl-EG as additive via electroless plating in the paper. As comparison, the different additive concentration of choline chloride-ethylene glycol (ChCl-EG) ionic liquid (0 g l^−1^, 30 g l^−1^, 60 g l^−1^, 90 g l^−1^) is presented. The effects of ChCl-EG concentration are studied, including the surface morphologies, phase analysis of Ni-decorated h-BN powders and the residual Ni^2+^ concentration is measured in electroless plating bath. It is demonstrated that the deposition phenomena of nickel particles on h-BN surface is changed with the addition of ChCl-EG. When the concentration of ChCl-EG is 30 g l^−1^, the Ni particles on h-BN surface are in dispersed and spheroid state with the average size of 10–1000 nm. It can be found that 30 g l^−1^ ChCl-EG is conducive to the arise of deposition phenomena, which is the formation of the single nickel particle on h-BN surface. Besides, more Ni particles are deposited on h-BN surface with the increase of nickel plating times, which is characterized with scanning electron microscope and transmission electron microscope. Furthermore, the deposition phenomenon and growth mechanism are proposed without and with ChCl-EG as additive to further elaborate the formation of Ni particles on h-BN surface.

## 1. Introduction

Electroless deposition process is an autocatalytic chemical reaction in aqueous solution which is used in surface metallization of conductive or non-conductive substrates, the extensive and potential surface engineering has been much faster developed [1–4]. The potential technique can be related to its unique quality of surface modification, such as the hardness, wettability, corrosion properties and the capability to generate distributed deposition on different materials [5–7]. Comparing with the conventional electroplating process, the electroless deposition technique offers impressive advantages in terms of lower processing cost because the electricity is not involved. It is possible for getting deposition on materials with the excellent properties by adjusting of the composition, the pH and the temperature of an electroless bath [8–10]. Over the years, the various micro and nano hard particles (SiC, TiC, WC, Cr_3_C_2_, Al_2_O_3_, etc.) have been successfully coprecipitated by the electroless deposition process to surface modification [6,11–19]. Otherwise, molybdenum disulfide [20–23], polytetrafluoroethylene [24–26], graphite [10,11,27] and carbon nanotubes [28,29] as soft lubricating particles have been developed to get self-lubricating electroless composite materials. Hexagonal boron nitride (h-BN) is another excellent high-temperature solid lubricant with the layered structure analogous to MoS_2_ and graphite [10,26], the solid lubricant is a promising alternative candidate material in special environment [30–32]. Nevertheless, some relevant reports reveal that h-BN has been relatively the least explored in tribology owing to its poor wettability with ceramic/metal matrix and its inadequate thermo-oxidative performance [5,31,33]. Li *et al*. revealed the incorporation of h-BN lubricant powders within matrix layer of active metal, as nickel could be used to improve the wettability performance of h-BN, and they had shown the optimal process of nickel-coated boron nitride [34–36]. Zhao *et al*. employed the hydrothermal hydrogen reduction to fabricate nickel-coated hexagonal BN particles [35,36]. Besides, Du *et al*. indicated the use of Ni-coated h-BN for laser treated composite coating [5,30]. Thus, detailed insights of depositing nickel particles on h-BN surface via the electroless deposition become an interesting problem.

Electroless deposition process has been explored in order to deposit nickel particles on h-BN surface. However, the instability of electroless plating bath limits the application of the process, because the Ni ions cannot be effectively used and they are easy to be deposited with the cluster in the electroless bath [16,37]. To overcome these shortcomings, a new kind of plating solution or additive needs to be found for the process. Recently, there has been a growing interest of the new class of substance with low melting point and vapour pressure, good conductivity, non-flammability and wide electrochemical window, named as room temperature ionic liquids [38]. Up to now, lots of studies have been reported on the application of ionic liquid as additives, such as lubricant additives [39,40], catalytic reactions and organics, and mobile-phase additives in liquid chromatography [41,42]. The research indicated that the addition of ionic liquids can effectively relieve the harmful effects of depositing quality and impurities. Therefore, Ru *et al*. fabricated Ni-decorated Al_2_O_3_ powders with [bmim] ionic liquid as additive, decreasing Ni clusters on the surface of Al_2_O_3_ powders and electroless plating bath. It is optimized that Ni particles are absolutely and uniformly deposited on the surface of powders [2,5,6].

In this paper, Ni-decorated h-BN powders are fabricated by electroless deposition from sulfate solution with ChCl-EG ionic liquid as additive. The effects of ChCl-EG concentration are studied, including the surface morphologies, the residual Ni^2+^ concentration is measured in electroless plating bath and the element distribution of Ni-decorated h-BN powders. Besides, the morphologies of coated products obtained with different nickel plating times are investigated. Based on the experimental results, an empirical model of deposition phenomenon without and with ChCl-EG as additive is proposed to further elaborate the formation mechanism of Ni-decorated h-BN powders.

## 2. Material and methods

### 2.1. Chemicals

**Figure 1.**
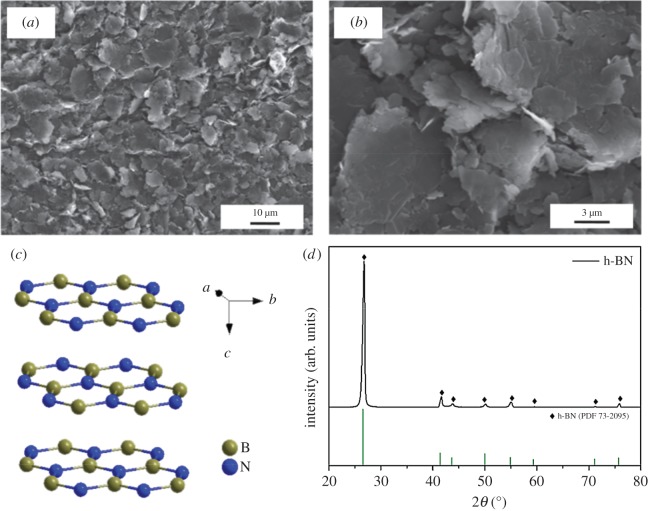
(*a,b*) SEM micrograph, (*c*) crystal structure and (*d*) XRD pattern of h-BN powders.

All the chemicals used in this work were purchased commercially with analytical grade (purity > 99.90%) from Chemical Reagent Co., Shanghai, China. Micrometre-sized flaky h-BN powder with a mean particle size of about 1–30 µm and the crystal structure of h-BN particle were supplied, as illustrated in figure 1.

### 2.2. Synthesis of ChCl-EG ionic liquid

ChCl-EG ionic liquid was used as the additive in the electroless deposition of Ni-decorated h-BN powders. ChCl-EG ionic liquid was synthesized from choline chloride (ChCl) and ethylene glycol (EG) under a dry vacuum atmosphere [2,9,43], and then re-crystallized to form a new deep eutectic solution (DES). The ChCl-EG DES was fabricated by mixing ChCl and EG (the molar ratio was 1 : 2) together and stirring the mixed components at 353 K until a homogeneous, colourless liquid formed. The eutectic melt was dried under vacuum for 24 h at a temperature of 333 K and stored in a capped glass bottle.

### 2.3. Pretreatment of h-BN

The Ni-decorated h-BN powders were fabricated by pretreatment process and electroless deposition. At first, h-BN powders were washed in the mixing solution of acetone and ethanol (the molar ratio 1 : 2) with the ultrasound for 15 min to remove the surface impurities. Then, the h-BN powders were repeatedly washed with distilled water to neutral and obtained by precipitation and dried in a vacuum oven at 353 K [5,29].

### 2.4. Fabrication of Ni-decorated h-BN powders

The electroless deposition of Ni particles on h-BN surface was performed in an electroless solution with Ni^2+^. The composition and concentration of the materials used in the electroless nickel bath are presented in table 1. First, 25 g NiSO_4_·6H_2_O was dissolved with 100 ml deionized water under the mechanical stirring speed of 300 r.p.m. Similarly, 16.5 g NaH_2_PO_2_·H_2_O and 20 g C_6_H_5_Na_3_O_7_·2H_2_O can also be, respectively, dissolved with 100 ml deionized water under 300 r.p.m.; 12.5 g H_3_BO_3_ was dissolved with 200 ml deionized water under 300 r.p.m. and 40°C. Then, NaH_2_PO_2_·H_2_O solution, C_6_H_5_Na_3_O_7_·2H_2_O solution and H_3_BO_3_ solution was added into NiSO_4_·6H_2_O solution in turn under 500 r.p.m. A small amount of NaOH solution was added into the above mixture solution to adjust pH of 8–9. Thus, the electroless plating bath was obtained according to above steps; 5 g h-BN was added into the above 500 ml electroless plating bath. Following that ChCl-EG (0 g l^−1^, 30 g l^−1^, 60 g l^−1^, 90 g l^−1^) as additive were studied in the paper [5]. Besides, different nickel plating times (one time, three times, five times) were selected to investigate the deposition phenomenon of Ni particles on h-BN surface. All reactions were carried out with a mechanical stirring speed of 300 r.p.m. min^−1^ and 60°C. The Ni-decorated h-BN powders were characterized after washing with distilled water several times, filtrated and dried in a vacuum oven at 353 K for 6 h. The electroless deposition experiments were repeated three times to ensure the reproducibility.

**Table 1.** Chemical composition of the electroless plating bath and operating parameters.

role in bath or operating parameters	constituent	concentration or conditions
main salt	nickel sulfate (NiSO_4_·6H_2_O)	50 g l^−1^
reducing agent	sodium hypophosphite (NaH_2_PO_2_·H_2_O)	33 g l^−1^
complexing agent	tri-sodium citrate (C_6_H_5_Na_3_O_7_·2H_2_O)	40 g l^−1^
buffering agent	boric acid (H_3_BO_3_)	25 g l^−1^
pH adjuster	sodium hydroxide (NaOH)	to adjust pH
h-BN powder	—	10 g l^−1^
mechanical stirring	—	300 r.p.m.
temperature	—	333 K
additive	ionic liquid (ChCl-EG)	0–90 g l^−1^
pH	—	8–9

### 2.5. Measurements and characterization

The surface morphology and elemental compositions of Ni-decorated h-BN powders were characterized by scanning electron microscope (SEM: ZEISS EVO 18) equipped energy dispersive spectroscopy (EDS: Bruker Quantax 200). Besides, the microstructures and crystallinity of sample were investigated by high-resolution transmission electron microscope (HRTEM: JEOL JEM-2100F). The utilization of nickel ions in electroless plating bath was measured via the inductively coupled plasma optical emission spectrometer (ICPOES: Optima 8000). The phases of the products were analysed by XRD (Rigaku MiniFlex 600) with Cu K_α_-radiation at a scan rate of 10° min^−1^ in the range of 2*θ* = 20–80°.

## 3. Results and discussion

### 3.1. Effects of ChCl-EG additive on Ni-decorated h-BN powders

**Figure 2.**
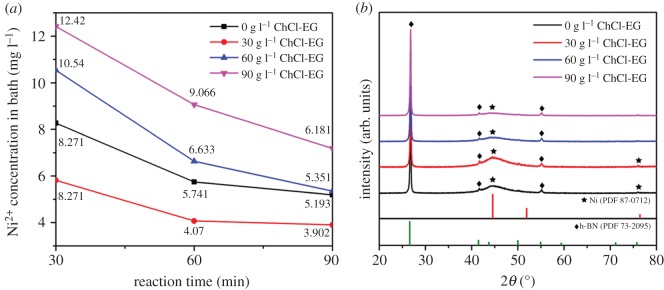
(*a*) The Ni^2+^ concentration in electroless solution in different reaction time, (*b*) XRD patterns of Ni-decorated h-BN powders fabricated with 0 g l^−1^, 30 g l^−1^, 60 g l^−1^ and 90 g l^−1^ ChCl-EG as additive.

In order to analyse the effect of ChCl-EG ionic liquid on the electroless deposition technique, the deposition experiments with different concentration of ChCl-EG are performed. Figure 2*a* is the residual Ni^2+^ concentration in electroless plating bath after different reaction time corresponding to the ChCl-EG concentration by the ICPOES analysis. It shows that the Ni^2+^ concentration in electroless plating bath is minimum with 30 g l^−1^ ChCl-EG as additive at any reaction time, this indicates that 30 g l^−1^ ChCl-EG ionic liquid is beneficial to deposit Ni particles from the solution. Besides, the XRD patterns of Ni-decorated h-BN powders fabricated with 0 g l^−1^, 30 g l^−1^, 60 g l^−1^, 90 g l^−1^ ChCl-EG as additive are indicated in figure 2*b*. It can be found that with the ChCl-EG concentration of 30 g l^−1^, the main phases of the samples are h-BN and metallic Ni, whereas the phosphorus is not observed, which may be below 4–5% and not characterized by XRD. The diffraction patterns of h-BN are in accordance with JCPDS card 73-2095 and that of Ni corresponds to JCPDS card 87-0712. The above results indicate that metallic Ni can be deposited from sulfate solution successfully when ChCl-EG ionic liquid is used as additive. Remarkably, the characteristic peaks of h-BN powders are distinct and sharp, but that of metallic Ni are weaker and broader with preferred orientation (111) plane, which can be ascribed to the poor crystallinity of metallic Ni. Besides, the XRD pattern of Ni-decorated h-BN powders obtained with 30 g l^−1^ ChCl-EG as additive is almost analogous to that of powders fabricated without ChCl-EG. However, the diffraction peaks of metallic Ni are much weaker when the addition of ChCl-EG is 60 g l^−1^ and 90 g l^−1^. It demonstrates the deposition of Ni grains cannot be affected with 30 g l^−1^ ChCl-EG as additive, conversely, it can be promoted in the reaction process, and this will be discussed in the following.

**Figure 3.**
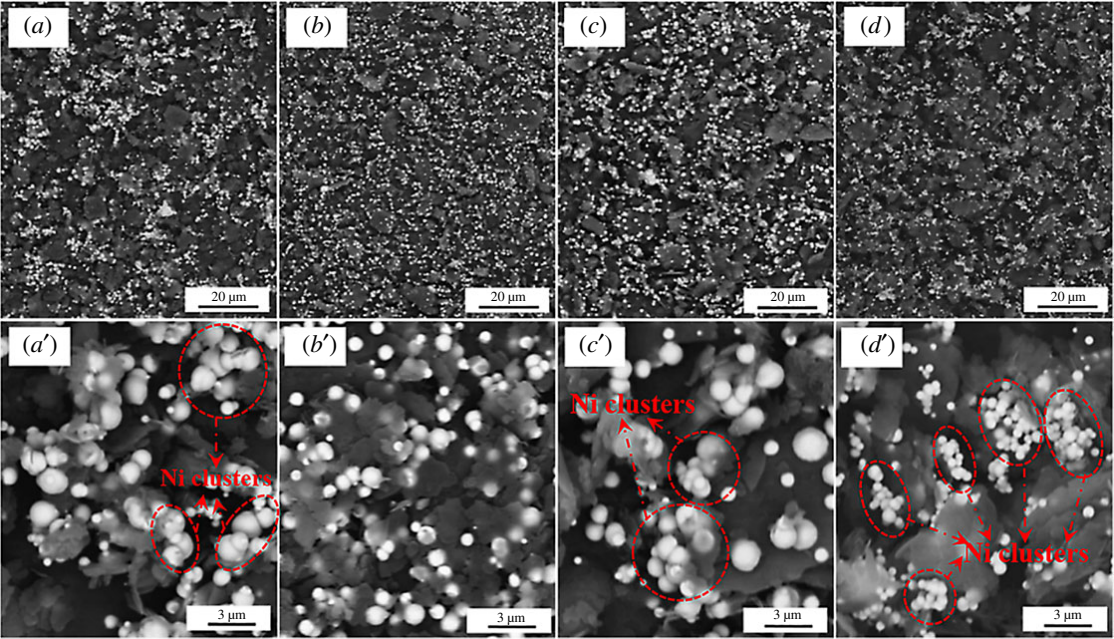
SEM morphologies of Ni-decorated h-BN powders in different concentration of ChCl-EG as additive (*a*,*a*′) 0 g l^−1^, (*b,b*′) 30 g l^−1^, (*c,c*′) 60 g l^−1^ and (*d,d*′) 90 g l^−1^.

**Figure 4.**
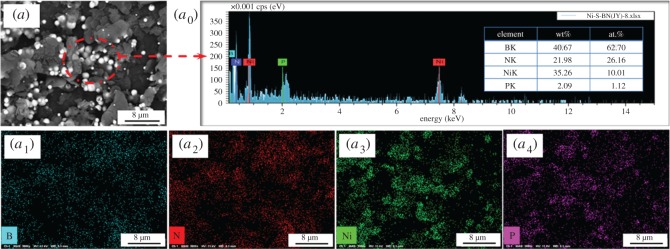
SEM morphologies (*a*) and EDS analysis (*a*_0_–*a*_4_) of Ni-decorated h-BN powders without ChCl-EG as additive.

The surface morphologies of Ni-decorated h-BN powders fabricated with different concentration of ChCl-EG are shown in figure 3*a–d*′. From figure 3*a*,*a*′, without ChCl-EG adding, there are many Ni clusters on the surface of h-BN, which are marked by the red dotted line, and the Ni particles are the uneven size. In addition, much Ni-free h-BN surface could be observed and small amounts of the independent Ni particles are scattered on h-BN powders. Those Ni particles are also spheroidal with a size of about 100–1500 nm. Accordingly, figure 4*a*_0_–*a*_4_ shows the element distribution of Ni-decorated h-BN powders fabricated without ChCl-EG as additive. There is also a strong Ni peak in the EDS spectrum. The element content of Ni and P are 10.01 at.% and 1.12 at.%, respectively. The content of Ni is slight lower than that of product obtained with 30 g l^−1^ ChCl-EG as additive while that of P is much the same. However, it can be seen that the many Ni clusters deposited on the surface of h-BN powders and most Ni particles with the uneven size are found from figure 3*a*,*a*′ and figure 4*a*.

When the ChCl-EG concentration is 30 g l^−1^, the Ni grains are deposited on the surface of flake h-BN powders and there hardly have the agglomerated Ni powders formed (figure 3*b,b*′), as mentioned in §3.2. With increase of ChCl-EG concentration to 60 g l^−1^, the Ni particles are distributed on the surface of h-BN, as is shown in figure 3*c–c*′, and the distribution is among the samples without ChCl-EG and with 30 g l^−1^ ChCl-EG as additive. Besides, a part of Ni clusters come into being and the size of Ni particles is inhomogeneous on h-BN powders with a size distribution of 50–2000 nm. At a higher ChCl-EG concentration of 90 g l^−1^ (figure 3*d,d*′), it is obviously seen that there are a lot of Ni clusters on h-BN powders and the size of Ni particles are much smaller, about 10–700 nm, which is nearly one-third as large as that of Ni particles in figure 3*c*′. Besides, the content of Ni is much less than the above three samples (0 g l^−1^, 30 g l^−1^, 60 g l^−1^ ChCl-EG as additive). Therefore, the metallic Ni in those samples detected is consistent with XRD examination (figure 2*b*). Based on the above comparative analysis, addition of 30 g l^−1^ ChCl-EG ionic liquid is optimum to obtain the Ni-decorated h-BN powders without Ni clusters on h-BN powders via electroless deposition.

### 3.2. Fabrication of Ni-decorated h-BN powders with 30 g l^−1^ ChCl-EG as optimum additive

**Figure 5.**
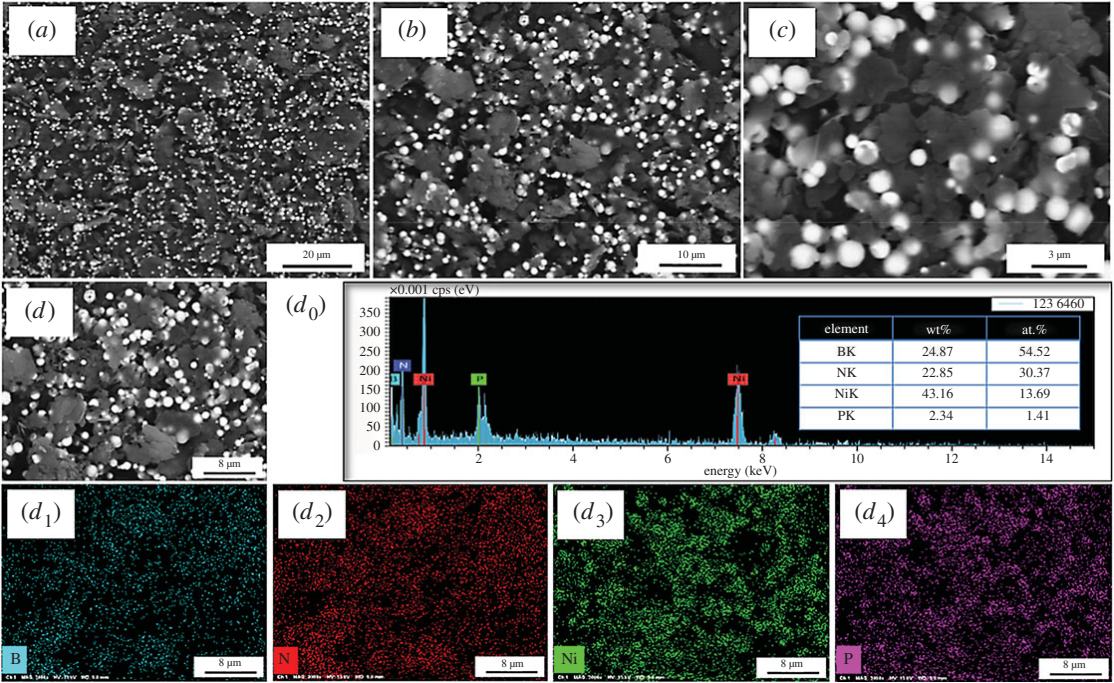
SEM images (*a–d*), EDS spectrum and mapping (*d*_0_–*d*_4_) of Ni-decorated h-BN powders fabricated with 30 g l^−1^ ChCl-EG as additive.

The SEM micrograph analysis of Ni-decorated h-BN powders fabricated with 30 g l^−1^ ChCl-EG as additive are illustrated in figure 5*a–c*. From figure 5*a*, the bright white Ni particles are deposited on the surface of flake h-BN powders and part of the h-BN surface is decorated by spheroidal Ni particles. A small amount of Ni clusters can be deposited on the surface of h-BN powders, and most Ni particles are in the dispersed state. As shown in figure 5*b*, the Ni particles are independently distributed on the surface of loosely accumulated flake h-BN powders. In addition, these little Ni particles are globular, with a mean size of about 10–1000 nm, and it can be also deposited on the region between layers, besides the surface of h-BN powders (figure 5*c*). Furthermore, in order to analyse the element distribution of Ni-decorated h-BN powders, the EDS is implemented as presented in figure 5*d*_0_–*d*_4_. The EDS spectrum shows a strong Ni peak corresponding to the flake h-BN powders, and the element content of Ni and P are 13.69 at.% and 1.41 at.%, respectively. From figure 5*d*_1_–*d*_4_, it can be seen that the Ni and P elements are distributed independently on the surface or between layers of h-BN powders. This distribution serving as the dispersed Ni particles forms a Ni-decorated structure.

**Figure 6.**
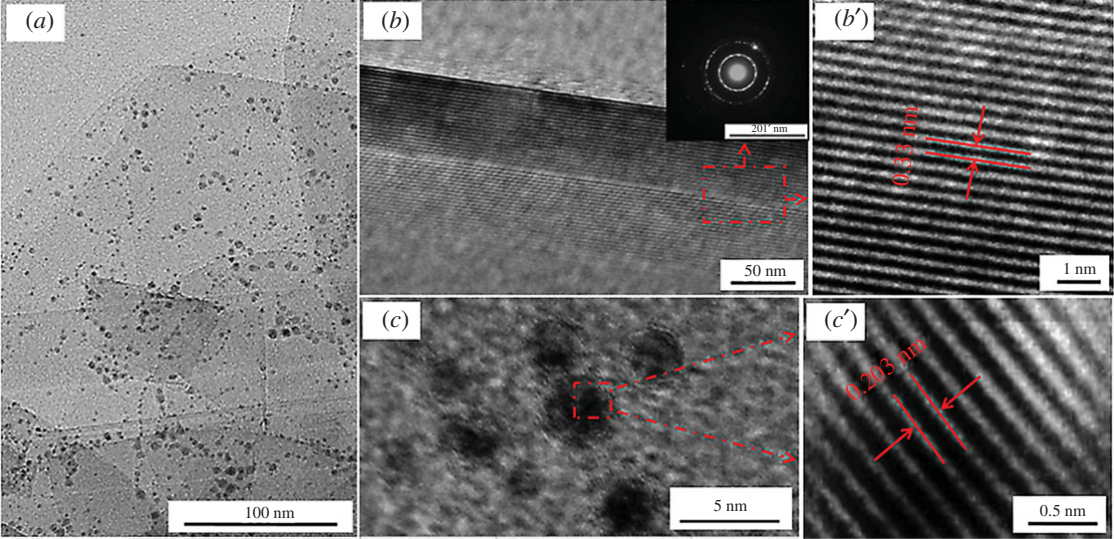
(*a*) TEM images of Ni-decorated h-BN powders with 30 g l^−1^ ChCl-EG as additive, (*b*,*b*′) HRTEM images and FFT analysis of h-BN powders (inset: corresponding SAED patterns), (*c,c*′) HRTEM images and FFT analysis of Ni grains on h-BN surface.

TEM and HRTEM analyses were carried out to get more detailed information about morphology and crystallinity of Ni-decorated h-BN powders. As shown in figure 6*a*, there are a number of nano-sized dark particles on the surface of laminar material through TEM images. Figure 6*b* is the HRTEM image of the laminar h-BN powder. The Fourier transform is obtained in the area of red square, as is presented in figure 6*b*′, the lattice fringes are clearly visible with a spacing of 0.33 nm, which is in good agreement with the spacing of the (002) plane of the crystal h-BN. The inset of figure 6*b* distinctly depicts the selected area electron diffraction (SAED) pattern of the red region; those diffraction rings are, respectively, corresponding to the crystal planes (011), (112), (006), (202) and (026). The high-index crystal faces are observed mainly owing to the relatively thin surface and the poor crystallinity of h-BN powders, but it is enough to prove that the sheet materials are h-BN powders. In order to verify the nano-sized dark little grains as Ni globules, the HRTEM image is acquired by figure 6*a*, as shown in figure 6*c*. Accordingly, the lattice fringes of dark little particles in red square are obtained by inverse Fourier transform (figure 3*b*′); it can be examined that the value of spacing is 0.203 nm. This parameter is consistent with the crystal plane (111) of Ni element, so the nano-sized dark particles are Ni globules, which cannot be observed with SEM due to the tiny size.

### 3.3. Effects of electroless plating times on Ni-decorated h-BN powders

**Figure 7.**
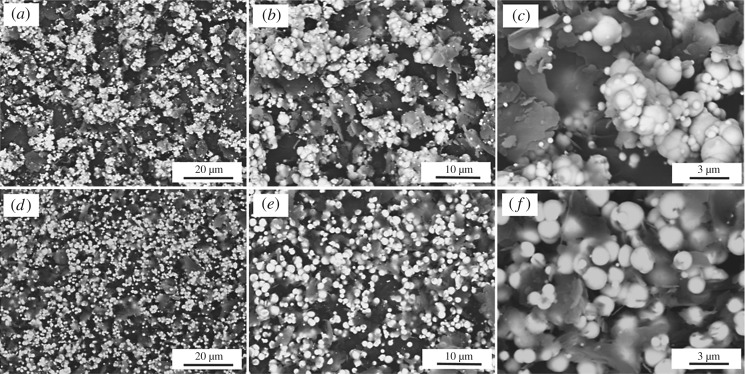
SEM micrographs of Ni-decorated h-BN powders with electroless plating three times: (*a–c*) without 30 g l^−1^ ChCl-EG and (*d–f*) with 30 g l^−1^ ChCl-EG as additive.

**Figure 8.**
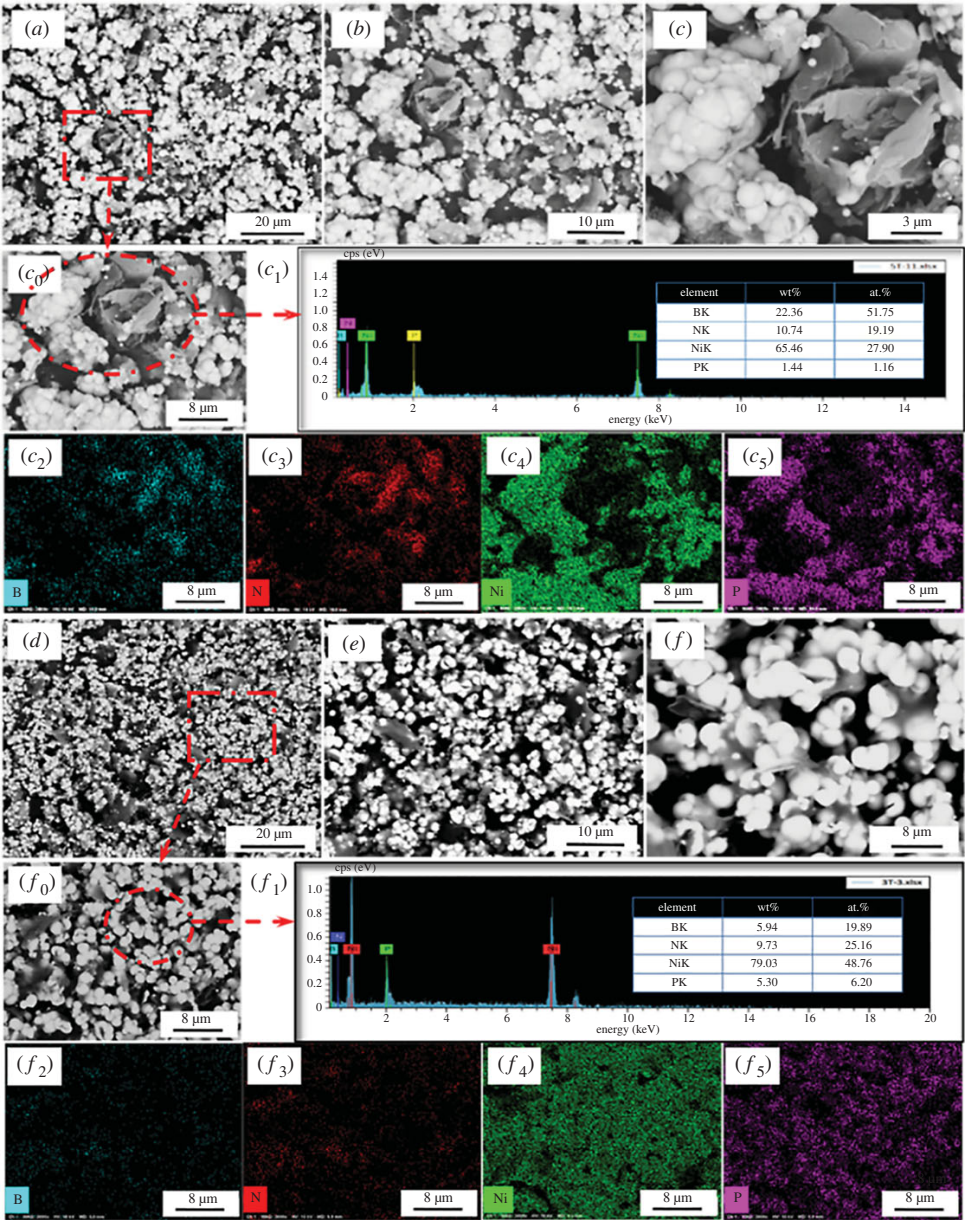
SEM micrographs and EDS analysis of Ni-decorated h-BN powders: (*a*–*c*_0_) SEM micrographs and (*c*_1_–*c*_5_) EDS analysis of plating five times without ChCl-EG, (*d*–*f*_0_) SEM micrographs and (*f*_1_–*f*_5_) EDS analysis of plating five times with 30 g l^−1^ ChCl-EG as additive.

To better analyse the deposition phenomena about Ni particles on the surface of h-BN powders, the fabrication of Ni-decorated h-BN powders is carried out without and with 30 g l^−1^ ChCl-EG as additive with one (figures 4 and 5), three (figure 7), five (figure 8) times of electroless deposition. When the Ni-decorated h-BN powders are obtained without ChCl-EG via the electroless plating one time, as shown in figure 4, little Ni particles are deposited on h-BN surface. It can be seen that a part of Ni clusters (figure 4*a* and *a*_3_). However, when the Ni-decorated h-BN powders are fabricated with 30 g l^−1^ ChCl-EG via the electroless plating one time, as presented in figure 5, the distribution of Ni particles was in independently and dispersed state on h-BN powders. Moreover, those Ni particles are globular with a mean size of about 10–1000 µm (from figure 5*b–d* and *d*_3_).

When the reaction times are extended to three times without ChCl-EG, as shown in figure 7*a–c*, many Ni clusters are deposited on h-BN surface and most h-BN are exposed. It indicates that most Ni particles are agglomerated without ChCl-EG as additive in the electroless bath and on the h-BN surface. While the Ni particles are uniformly and dispersedly deposited on h-BN surface with ChCl-EG as additive via plating three times, as shown in figure 7*d–f*. Thus, it is indicated that ChCl-EG as additive can effectively hinder the agglomeration of Ni particles on h-BN surface. However, it can be also seen that a small part of h-BN surface is not deposited by Ni particles (figure 7*f*).

Then, the reaction times are increased to five times in figure 8. As presented in figure 8*a–c*, it can be seen that more Ni clusters are deposited on h-BN surface and those clusters become much bigger. Besides, there is also much exposed h-BN surface. The deposition phenomenon is analogous to figure 7*a–c*. The EDS spectrum shows the element content of Ni and P are 27.90 at.% and 1.16 at.%, respectively. It indicates that more and more Ni particles are deposited on h-BN surface. Besides, it is obvious that the deposited Ni particles are in stacked state from figure 8*c*_0_ and *c*_4._ As reaction times extended to five times with ChCl-EG as additive, as shown in figure 8*d–f*, the Ni particles are dispersedly and uniformly deposited on the surface of h-BN, compared with figure 7*a–c*. The EDS analysis indicates that the element content of Ni and P are 48.76 at.% and 6.20 at.%, respectively. The distribution of Ni particles is also dispersive, as shown in figure 8*f*_0_–*f*_5._ It demonstrates ChCl-EG as additive is beneficial to deposit Ni particles on h-BN surface. The phenomenon is consistent with figure 2*a*. Besides, Ni particles are compactly deposited on the surface of h-BN powders from figure 8*d*–*f*_0_.

**Figure 9.**
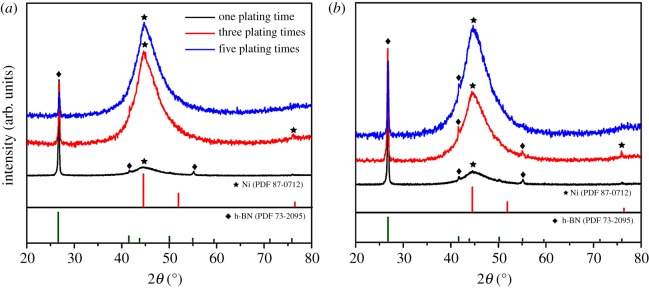
XRD pattern of Ni-decorated h-BN powders via the electroless plating one, three, five times: (*a*) without ChCl-EG, (*b*) with 30 g l^−1^ ChCl-EG as additive.

The XRD patterns of Ni-decorated h-BN powders fabricated without and with ChCl-EG as additive via the electroless plating one, three, five times are illustrated in figure 9. From figure 9*a*, it can be seen that the peak of metallic Ni is much stronger and broader at 2*θ* = 44.50° when the reaction times are extended from plating three times to five times. The JCPDS cards of Ni and P elements are consistent with the mentioned contents in §3.1. Remarkably, the main peaks of h-BN are distinct and sharp at 2*θ* = 26.74°, being analogous to the figure 2*b*, while the other peaks of h-BN can be weakened or disappear owing to the increasing content of metallic Ni, which is in agreement with the SEM micrographs without ChCl-EG. The Ni-decorated h-BN powders are fabricated with ChCl-EG as additive, as shown in figure 9*b*. With the increase of plating times, the chance of Ni element and h-BN powders peak is analogous to figure 9*b*. It indicates that more Ni particles are deposited with the increased plating times, which is consistent with the textual SEM micrographs. From figure 9, it can be demonstrated that more and more Ni particles are deposited from sulfate solution. Therefore, the Ni-decorated h-BN structure can be obtained by the deposition of metallic Ni on h-BN powders with increasing electroless plating times. Accordingly, the electroless process can be explained by the following possible deposition phenomena and growth mechanism.

### 3.4. Deposition phenomena and growth mechanism of Ni-decorated h-BN powders

**Figure 10.**
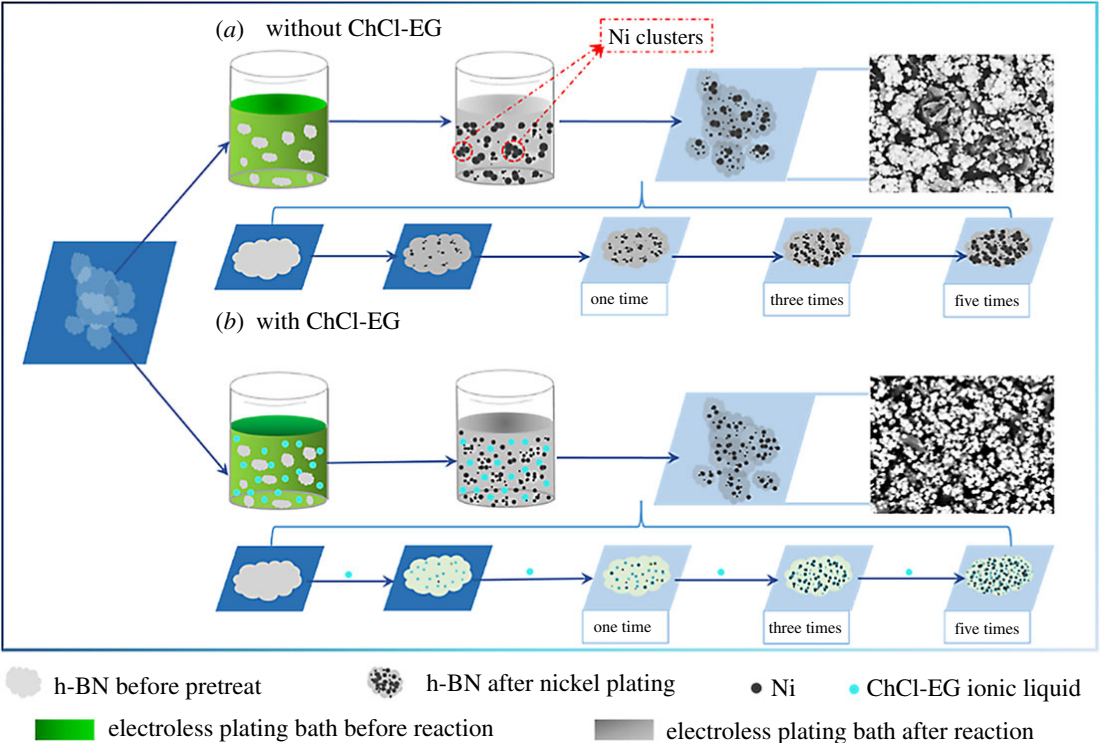
Electroless deposition phenomena of Ni-decorated h-BN powders: (*a*) without ChCl-EG and (*b*) with 30 g l^−1^ ChCl-EG ionic liquid as additive.

Based on the above analysis and the relevant reference [2,44], the possible deposition phenomena and growth mechanism of Ni particles on h-BN powders without and with ChCl-EG as additive are proposed respectively, as shown in figure 10. After the pretreatment process of h-BN, the surface impurities are removed to deposit Ni particles on the h-BN surface. When the cleaned h-BN powders are introduced into the electroless plating solution without ChCl-EG, the Ni^2+^ and P^+^ are reduced respectively into Ni and P elements by hypophosphite with the condition of temperature and stirring [33,44]. Meanwhile, the colour of the solution is turned from green to light green with little grey and a great deal of hydrogen bubbles appear together, indicating the deposition of Ni metallic with P element from the solution, as shown in equations (3.1) and (3.2). The standard free energy is the negative value, so the reaction of reducing Ni^2+^ is spontaneous [33,36,45]. Meanwhile, those energies of temperature and mechanical stirring speed were implemented to speed up the reaction.

The nano-sized Ni particles with high surface area and energy are generated in the electroless bath and on the h-BN surface, as presented in figure 10*a*. Besides, the Ni particles are easy to being the cluster on the h-BN surface powders and in the electroless solution. It is a result that the Ni particles are more inclined to form a nucleus and grow on the original nucleation point, which is the aggregative place of Ni particle after the electroless plating one time. With the increase of plating times, the formed Ni clusters of plating one time will lead to more agglomerated state of Ni particles on the surface of h-BN powders. Meanwhile, many independently separated Ni clusters away from the h-BN surface develop the irregular dendrites and blocks, as shown in the illustration of figure 10*a*.

3.1 $$\begin{array}{rl} {\text{Ni}}^{2+}+{\text{H}}_{2}\text{P}{{\text{O}}_{2}}^{-}+3\text{O}{\text{H}}^{-} & \to \text{HP}{{\text{O}}_{3}}^{2-}+\text{N}{\text{i}}^{0}(\text{s})+2{\text{H}}_{2}\text{O}\\ (\Delta_{r}G^{\theta } & =-258.62\;\text{kJ}\;\text{mo}{\text{l}}^{-1}) \end{array}\}$$

and

3.2 $$\begin{array}{rl} {\left[\text{Ni}{\left(\text{Cit}\right)}^{3-}\right]}^{-}+{\text{H}}_{2}\text{P}{{\text{O}}_{2}}^{2-}+3\text{O}{\text{H}}^{-} & \to \text{N}{\text{i}}^{0}(\text{s})+\text{Ci}{\text{t}}^{3-}+\text{HP}{{\text{O}}_{3}}^{2-}+2{\text{H}}_{2}\text{O}\\ (\Delta_{r}G^{\theta } & =-213.60\;\text{kJ}\;{\text{mol}}^{-1}) \end{array}\}$$

When ChCl-EG ionic liquid is used as additive, the electroless deposition process of Ni particles on the surface of h-BN powders is extraordinarily different, as illustrated in figure 10*b*. The Ni particles are easy to be dispersedly and uniformly precipitated on the surface of flake h-BN powders, owing to the nucleation point may appear on the hollow surface of h-BN powders during the process. Meanwhile, the ChCl-EG additive efficiently hinders the random nucleation and agglomeration of Ni grains. This is mainly because some regions result in the adsorption of ChCl-EG, so the distribution of Ni particles was relatively homogeneous via the electroless plating one time (figure 10*b*).

Most regions of h-BN surface are covered by Ni particles with the increase of plating times, which is mainly because Ni particles are easy to nucleate and grow on the formed Ni globules of the first electroless plating, as is shown in the illustration of figure 10*a*. When the reaction was beginning, some tiny Ni particles deposited randomly on the surface of h-BN powders. As the reaction proceeding, a part of Ni particles is gradually growing on the original site, and those particles are bigger and bigger. Besides, some new Ni particles nucleation points can be found on h-BN surface. The Ni-decorated h-BN powders are fabricated with ChCl-EG by plating one time, but the Ni particles are dispersedly deposited with less number. In order to deposit uniformly Ni particles on the surface of h-BN, the electroless plating times were extended to three times and five times. Correspondingly, the Ni particles are compactly and uniformly deposited on the surface of h-BN powders, as can be presented from figure 8*f* and the illustration of figure 10*b*. Hence, some dispersedly and compactly Ni-decorated h-BN powders can be obtained via the electroless plating with ChCl-EG ionic liquid as additive.

## 4. Conclusion

(1) The Ni particles are more intended to be dispersedly deposited on h-BN surface from 0 to 30 g l^−1^ ChCl-EG ionic liquid. Then, when the ChCl-EG additive is increased from 30 to 90 g l^−1^, the distribution of Ni particles on h-BN surface comes gradually into being the aggregated state and have the tiny size. Therefore, the addition of 30 g l^−1^ ChCl-EG ionic liquid is optimum to obtain the independently and uniformly Ni particles on the h-BN surface by electroless deposition.
(2) The deposited Ni particles from sulfate solution can be distributed on the h-BN powders by electroless deposition with 30 g l^−1^ ChCl-EG ionic liquid as additive. The Ni particles on h-BN surface are spheroidal with a mean size 10–1000 nm.
(3) The deposition phenomena of Ni-decorated h-BN powders are studied with the increase of the electroless plating times based on the changes in morphology. Ni clusters are more and more without ChCl-EG. Conversely, Ni particles can be dispersedly and uniformly on h-BN surface with ChCl-EG as additive.
(4) The deposition phenomena and growth mechanism of Ni-decorated h-BN powders are put forward. The mechanism emphasizes that 30 g l^−1^ ChCl-EG as additive is more prone to form the dispersed and uniform Ni particles on the surface of h-BN powders to gain the Ni-decorated h-BN powders without Ni clusters.

## Data accessibility

The datasets supporting this article have been upload to Dryad Digital Repository: http://dx.doi.org/10.5061/dryad.f2579 [46].
